# P-229. Screening and Incidence of Sexually Transmitted Infections (STI) among Persons Living with HIV (PWH) in the Illinois Department of Corrections (IDOC)

**DOI:** 10.1093/ofid/ofaf695.451

**Published:** 2026-01-11

**Authors:** Daniel Schreiber, Emily N Drwiega, Miguel Perez, Rita Uda, Mahesh C Patel, Scott Borgetti, Melissa E Badowski

**Affiliations:** University of Illinois Hospital and Health Systems, Chicago, IL; University of Illinois Chicago, Chicago, Illinois; UIC Retzky College of Pharmacy, Chicago, Illinois; UIC Retzky College of Pharmacy, Chicago, Illinois; University of Illinois Chicago, Chicago, Illinois; University of Illinois at Chicago, Chicago, Illinois; University of Illinois Chicago, Chicago, Illinois

## Abstract

**Background:**

Sexually transmitted infections (STIs) have a disproportionately high prevalence in individuals in custody and persons with HIV (PWH). However, limited data exists for the rates of infection for individuals in custody, particularly PWH, leading to a potential gap in timely and appropriate recognition and treatment of STIs. In the Illinois Department of Correction (IDOC), persons in custody are screened at initial intake and those with HIV are seen by a multidisciplinary care team to manage HIV care and related STIs.

**Figure:**
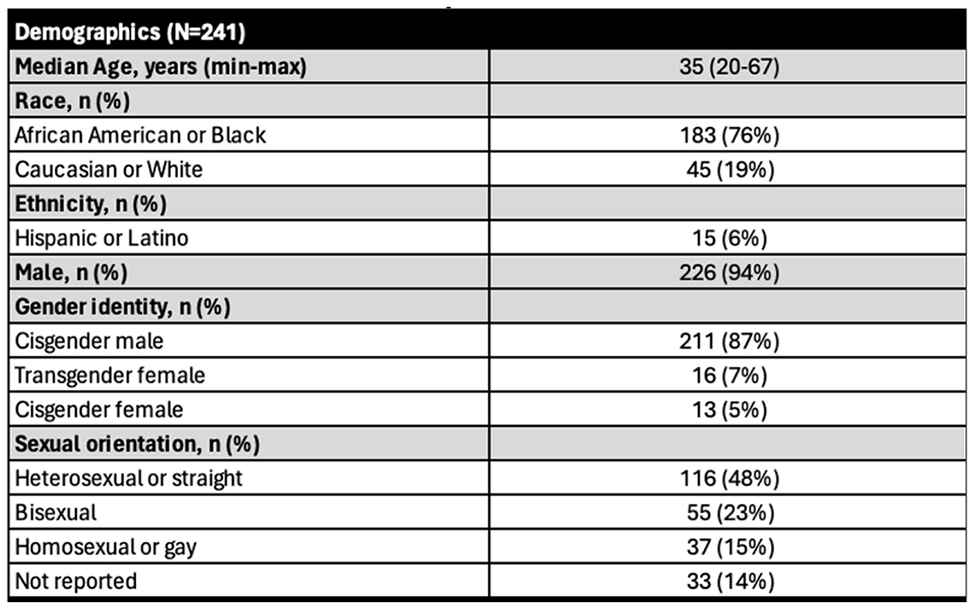
Persons with HIV in Illinois Department of Corrections Patient Demographics Of 241 patients with HIV in IDOC , the majority of patients were Black, cisgender males. Most patients were heterosexual with a median age of 35 years old. Demographic information was self-reported from the patient.

**Figure:**
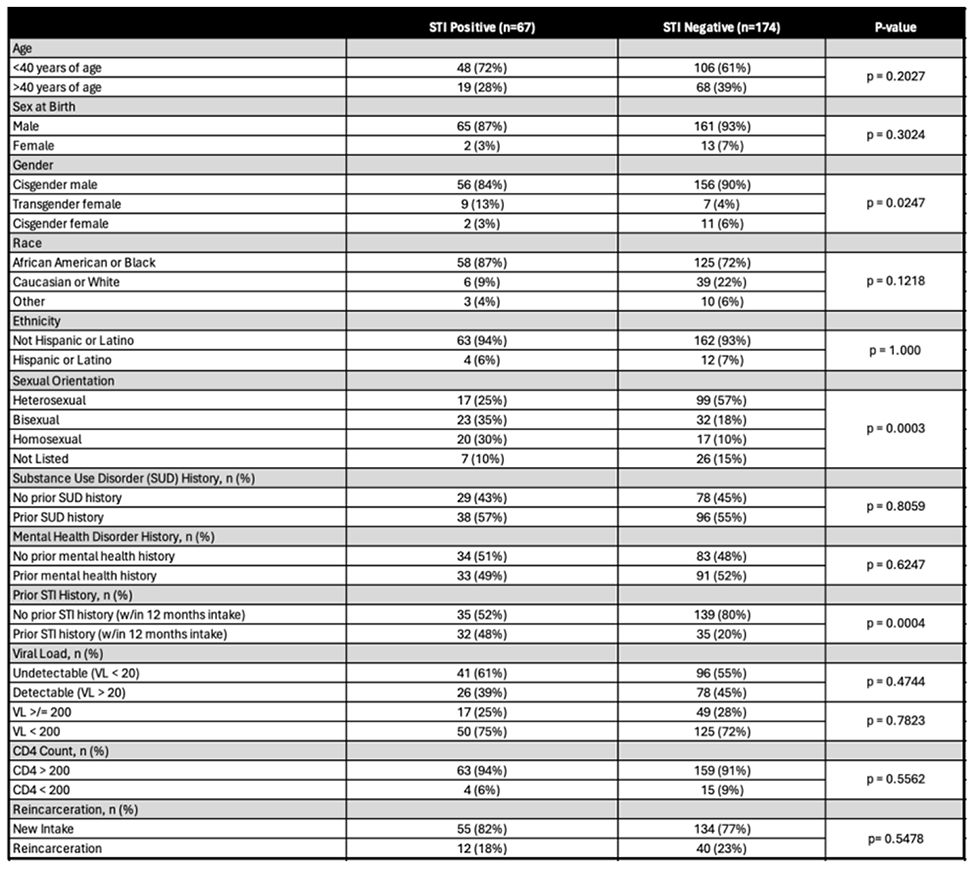
Predictors and Risk factors of STI Positivity Of 241 PWH in IDOC, 67 patients screened positive for at least one STI. STI Positive was a composite variable, and a patient was deemed positive if criteria were met for STI positivity for gonorrhea, chlamydia, or syphilis. Patients were considered an active/new infection for gonorrhea and chlamydia if first positive NAAT via urine in the system. Patients were considered positive for syphilis if one of the following were true: (a) first positive in system, (b) 4-fold higher than a previous titer, (c) positive titer followed a previously negative titer. Information and risk factors (mental health history, STI history, and substance use disorder history) was self-reported from the patient.

**Methods:**

Electronic medical records of PWH receiving care via IDOC telemedicine in conjunction with the University of Illinois Hospital and Health Sciences System were reviewed from January 1, 2021 to June 30, 2024. The primary objective was to determine frequency of screening and positivity for gonorrhea, chlamydia, and syphilis. HIV viral load and other known STI risk factors were also collected to assess predictors of STI positivity.

**Results:**

The majority of PWH in IDOC were Black, cisgender males (Table 1). Of 241 patients with HIV in IDOC, a total of 226 (94%), 98 (41%), and 97 (40%) patients were screened for syphilis, gonorrhea, and chlamydia, respectively. Of those screened, 218/226 (96%), 26/98 (27%), and 26/97 (27%) were screened for syphilis, gonorrhea, and chlamydia, respectively, at intake. Following linkage to care with the multidisciplinary telemedicine team, more patients were screened for gonorrhea (70/98, 88%), and chlamydia (71/97, 73%) compared to at intake. 65/226 (29%), 3/98 (3%), and 2/97 (2%) patients were positive for syphilis, gonorrhea, and chlamydia, respectively, with 41/65 (63%) representing new syphilis diagnoses. STI positivity was associated with women who were transgender (p=0.02), men who have sex with men (p< 0.001), and a history of STI (within 12 months prior of intake) (p< 0.001) (Table 2).

**Conclusion:**

The majority of patients were screened for syphilis during their time in custody and approximately 1 in 5 were new diagnoses. Gonorrhea and chlamydia were infrequently screened for with low rates of positivity. Routine screening for STIs for PWH by multidisciplinary care teams has the potential to identify a high proportion of STIs with key patient demographics and past medical history serving as potential predictors of STI positivity.

**Disclosures:**

Scott Borgetti, MD, GlaxoSmithKline: Grant/Research Support

